# On-Line Monitoring of Vitamin C in Fruit Juice in Processing Plants by Electrochemical Sensor Based on PEDOT-Modified Electrodes: A Feasibility Study

**DOI:** 10.3390/s25051385

**Published:** 2025-02-24

**Authors:** Chiara Giliberti, Matteo Malavasi, Simone Fortunati, Luca Cattani, Marco Giannetto, Sara Rainieri, Maria Careri

**Affiliations:** 1 Department of Chemistry, Life Sciences and Environmental Sustainability, University of Parma, Parco Area delle Scienze 157/A, 43124 Parma, Italy; chiara.giliberti@unipr.it (C.G.); simone.fortunati@unipr.it (S.F.); maria.careri@unipr.it (M.C.); 2 Department of Engineering and Architecture, University of Parma, Parco Area delle Scienze 181/A, 43124 Parma, Italy; matteo.malavasi@unipr.it; 3 Department of Engineering for Industrial Systems and Technologies, University of Parma, Parco Area delle Scienze 181/A, 43124 Parma, Italy; luca.cattani1@unipr.it

**Keywords:** electrochemical sensors, gold nanoparticles, PEDOT, vitamin C on-line measurement, on-line monitoring

## Abstract

Vitamin C, an antioxidant in most fruits and vegetables, is highly sensitive to heat, pH, metals, light, and oxidation, making it a key marker for nutrient degradation in thermal processing. Research aimed at improving processing methods to maximize vitamin C retention is usually limited to expensive laboratory equipment, which does not reflect real-world conditions in the food industry. On the other hand, traditional methods are not suitable for on-line monitoring. This paper proposes bridging the gap in liquid food processing with a voltammetric sensor based on poly(3,4-ethylenedioxythiophene)-modified screen-printed carbon electrodes. The sensor showed excellent repeatability, with intra-sensor RSD below 5% and inter-sensor RSD below 10% at 250 mg/L of ascorbic acid. Detection and quantification limits were 0.7 and 2.1 mg/L, respectively. Trueness assessment in commercial orange juice with a declared vitamin C content yielded a recovery rate of 94 ± 1%. Selectivity tests with citric acid at concentrations equal to and 20 times higher than that of ascorbic acid showed no significant interference. Shelf-life studies confirmed the stability of the sensor for at least two months. This nanocomposite-based approach balances performance and cost with simple preparation, affordable materials, and a stable coating that allows long-term storage in uncontrolled environments.

## 1. Introduction

L-ascorbic acid (C_6_H_8_O_6_), commonly known as ascorbic acid (AA), has the chemical name *2-oxo-L-threo-hexono-1,4-lactone-2,3-endiol*. AA is a crucial water-soluble vitamin essential for the biosynthesis of collagen, carnitine, and neurotransmitters [1]. Primarily found in fresh fruits and vegetables, vitamin C is abundant in oranges, lemons, grapefruits, tangerines, red fruits, raspberries, blackcurrants, and redcurrants. However, it degrades easily during storage, food processing, and heat treatment, leading to significant quality changes in fruits, vegetables, and juices, ultimately limiting their nutritional value and shelf-life. Due to its high biodegradability, retaining vitamin C during food processing can be quite challenging. Because of this, AA can be used as a quality marker to indicate the preservation of other more resilient nutrients and compounds during food processing and storage [1,2,3,4,5].

Accurate modeling of the behavior of AA during blanching, drying, cooking, and storage is necessary to optimize these processes. Among all vitamins, vitamin C is the least stable and is quickly degraded, influenced by factors such as pH [6,7], temperature [8,9], light exposure [10,11], and the presence of oxygen, enzymes [6], hydrogen peroxide [11], and metallic catalysts [12,13,14]. Depending on these factors, degradation can occur either aerobically or anaerobically. Anaerobic degradation is complex and relatively insignificant in most foods, while aerobic conditions oxidize ascorbic acid to dehydroascorbic acid, followed by hydrolysis and further oxidation [15]. Primary degradation products include furfural, 3-hydroxy-2-pyrone (3H2P), and furoic acid, although other compounds may form based on reaction conditions.

Researchers are working to optimize industrial processing methods and conditions to maximize vitamin C retention. Understanding the degradation mechanisms of vitamin C under different processing conditions will aid in controlling parameters for better preservation, guaranteeing a nutritionally superior quality product. Additionally, understanding degradation kinetics and various kinetic models is crucial for predicting vitamin C loss and quality changes under specific processing conditions. However, most research on the thermal breakdown of vitamin C has focused exclusively on laboratory testing, which does not fully reproduce the practical conditions in the food industry. In complete industrial-scale processing lines, variations in the nutritional and quality attributes of the final product, compared to the raw ingredients, can result from numerous factors and processes occurring before or after the heat treatment stage. For instance, as shown in [16], in chunky fruit desserts prepared from apple puree, the formation of 3H2P and furoic acid was primarily due to oxidative degradation of AA rather than thermal breakdown. For these reasons, continuous monitoring of vitamin C content throughout the entire production process would be highly valuable in identifying and addressing critical points in the plant. Although conventional analytical techniques, such as high-performance liquid chromatography, allow the determination of ascorbic acid [17] with the potential of the determination of both the reduced and oxidized forms (ascorbic and dehydroascorbic acids) of vitamin C and the simultaneous separation of different water-soluble vitamins [18], they require specialized laboratory equipment and time-consuming sample treatment procedures that preclude their use for continuous in-process monitoring to control processes in real time. Conversely, sensors exhibit promising potential as in-process analyzers for Process Analytical Technology (PAT) to be implemented and used in the food industry for timely decision-making during production because they allow rapid measurements and require minimal or no sample preparation [19]. On the other hand, the reliability of the measurement results must be demonstrated in real-life conditions to effectively implement these PAT strategies in the food industry [19]. Even though many promising scientific studies deal with the development of electrochemical sensors, they are difficult to extend to be applied as in-line/on-line analyzers for on-site food quality control due to the complex sample handling and the cost of sensor material [20,21,22,23,24,25,26]. Biosensors are also widely applied for AA determination in a variety of areas, including food quality monitoring; however, even though biosensing devices are rapid, simple, economical, and extremely sensitive, electrochemical ascorbate biosensors suffer from a short shelf-life and from a narrow temperature range [27]. Biosensors are also not suitable in the context of PAT applications.

In such a context, this paper proposes a solution to bridge this gap in liquid food processing plants: a voltammetric sensor based on screen-printed carbon electrodes (SPCEs) modified by electrodeposition of gold nanoparticles (GNP) followed by electropolymerization of poly(3,4-Ethylenedioxythiophene) (PEDOT) is exploited, offering a major benefit in its ease of measurement and straightforward integration into various sections of the industrial plant thanks to the affordability of the device. As part of the research activities carried out within the project “Strengthening of the Italian Research Infrastructure for Metrology and Open Access Data in support to the Agrifood” (METROFOOD-IT), the present study aims to highlight the importance of specifically designing production lines to enhance vitamin C preservation in thermal processing at an industrial scale using in-process measurement systems. In this feasibility study, the primary objective is to validate the GNP/PEDOT nanocomposite-based sensor for future applications of monitoring vitamin C in fruit juices at an industrial scale to demonstrate a fit-for-purpose process analytical strategy capable of providing reliable quality data even under routine plant operating conditions and with negligible intervention from operators and experts. The ultimate goal is to measure critical control parameters designed to ensure that critical quality attributes fall within the desired specification limits of the final product.

## 2. Materials and Methods

### 2.1. Materials and Chemicals

Sodium chloride (NaCl), disodium hydrogen phosphate (Na_2_HPO_4_), hydrochloric acid (HCl, 37%), sodium hydroxide (NaOH), lithium perchlorate (LiClO_4_), 3,4-ethylenedioxythiophene (EDOT), potassium chloride (KCl), potassium dihydrogen phosphate (KH_2_PO_4_), potassium ferrocyanide (K_4_[Fe(CN)_6_]), chloroauric acid (HAuCl_4_), ascorbic acid, and citric acid were purchased from Sigma-Aldrich (Milan, Italy) The composition of Phosphate-Buffered Saline (PBS) was 137 mM NaCl, 2.7 mM KCl, 1.2 mM KH_2_PO_4_, and 8 mM Na_2_HPO_4_ (pH adjusted to 7.4 using HCl).

Ultrapure water (18.2 MΩ cm) was obtained from a Direct Q^®^ 3 UV ultrapure water system.

The standard solutions were prepared by dissolving ascorbic acid in PBS buffer. Screen-printed Carbon Electrodes DRP-C110 (SPCE DRP-C110) with 0.2 cm working electrode radius, carbon counter electrode, and silver pseudo-reference electrode were purchased from Metrohm Italiana Srl (Origgio, Varese, Italy).

As for the potential of the pseudo-reference electrode of SPCEs, 1 mM K_4_[Fe(CN)_6_] solution, prepared in PBS, exhibited an E_1/2_ potential of +0.01 V relative to the silver screen-printed electrode.

### 2.2. Equipment

The modification of SPCEs and the voltammetric readout were carried out using a Multi Autolab M204 10-channel potentiostat/galvanostat (Metrohm Italiana Srl, Origgio, Varese, Italy), which was operated via NOVA 2.1.6 version number Advanced Electrochemical Software. The SPCEs were connected to a connector box from DropSens (Origgio, Varese, Italy; DRP-DSC), which enabled their interfacing with the potentiostat.

A T-type thermocouple (TERSID, Sesto San Giovanni, Milano, Italy) was inserted into one sample to monitor temperature. The start of the treatment period commenced once the internal temperature reached the target level. Temperature readings were obtained using a data acquisition system (National Instruments NI CDAQ chassis with three NI 9213 C Series modules, National Instruments Italy S.r.l., Assago, Milano, Italy) connected to a KAYE K170–274 ice point reference (Amphenol Advanced Sensors Germany GmbH, Pforzheim, Germany).

### 2.3. Sample Preparation and Storage

Fresh orange juice was produced by crushing fresh oranges; 50 mL aliquots were collected in PET Falcon tubes and stored at −80 °C.

For the thermal treatment, 15 mL PET Falcon tubes were used for pasteurization, which was carried out in a Julabo ED Heating Immersion Circulator water bath (Julabo Labortechnik GMBH, Seelbach, Germany). PET Falcon tubes were chosen for their ability to withstand the explored temperature range (70–90 °C) while preserving the quality of the contents. The juice sample was filled to eliminate headspace, and the containers were securely sealed to prevent any evaporation. After completion of the treatment, samples were collected in triplicate and analyzed following a period of cooling at room temperature (20–26 °C), after which a 1:1 dilution with PBS buffer was undertaken.

### 2.4. Sensor Preparation and Voltammetric Determination of Vitamin C

The surface of SPCEs was functionalized with GNP and PEDOT. For the first functionalization layer, GNPs were electrodeposited under potentiostatic conditions by drop casting 50 µL of 5 mM HAuCl_4_ in 100 mM KNO_3_ aqueous solution onto the surface of SPCEs and applying a potential of −1.6 V for 200 s, at room temperature, ranging from 20 to 26 °C. Following the electrodeposition of GNPs, the electrodes were subjected to a two-step activation process. This involved dipping the electrodes in a solution of 1.2 M NaOH for 5 min and then immersing them in 1.2 M HCl for the same duration.

The GNP-modified SPCEs were then subjected to potentiostatic electropolymerization of EDOT for further functionalization. To this aim, 50 µL of a 5 mM EDOT aqueous solution in 0.1 M LiClO_4_ were drop cast onto GNP-SPCEs, and a potential of +0.8 V was applied at room temperature, ranging from 20 to 26 °C. To control the thickness and the amount of the PEDOT layer, the electropolymerization process was stopped after a charge density of 20 mC·cm^−2^ was reached, according to the results of our previous study [28]. After each step of the process, the electrodes were rinsed with distilled water and dried under nitrogen.

For the determination of vitamin C in standard solutions and in orange juice, Differential Pulse Voltammetry (DPV) measurements were performed by scanning the potential from −0.5 to +0.6 V with a step potential of 0.006 V, a modulation amplitude of 0.05 V, a modulation time of 0.1 s and an interval time of 0.5 s. Prior to undertaking each subsequent measurement obtained from the same sensor, a preconditioning phase at −0.5 V for 30 s was introduced to regenerate the PEDOT redox mediator in its reduced form, which is active for the electrocatalytic oxidation of AA.

### 2.5. Analytical Validation of the Electrochemical Sensor

The validation of the developed electrochemical sensor was performed according to the Eurachem guidelines [29]. The limit of detection (LOD) was calculated as 3.3·*s*_0_/√n and the limit of quantification (LOQ) as 10·*s*_0_/√n, where *s*_0_ is the blank standard deviation measured on 10 replicated blanks and n is the number of replicate measurements. Precision in terms of intra-sensor and inter-sensor repeatability was evaluated from replicated measurements for each concentration level explored, as discussed in more detail in Section 3.2. The linearity of the sensor was assessed by a calibration curve in the range of 45 to 600 mg/L AA.

Quantitative determination of AA in fresh orange juice was carried out by the standard addition method by performing two consecutive additions of 2 mL of a 2000 mg/L standard solution to 8 mL of juice sample diluted 1:1.

Trueness was evaluated using the percent recovery rate (%RR), calculated as (found concentration/claimed concentration) × 100. The “found concentration” refers to the ascorbic acid concentration determined using the developed sensor in a commercial orange juice sample with a specified vitamin C content. The selectivity of the sensor was assessed by the DPV response of AA obtained in the presence of citric acid, as the major active interferent in a fruit juice, after the addition of a standard solution of citric acid at two concentration levels, i.e., at equal concentration and at concentration 20 times higher than that used for the additions of standard ascorbic acid.

The stability of the GNP/PEDOT-based sensor was investigated by evaluating the shelf-life of the nanocomposite sensing coating for up to 8 weeks after storage at room temperature, ranging from 20 to 26 °C. During the storage period, the DPV signal from a freshly prepared 250 mg/L standard solution of AA was acquired at regular seven-day intervals.

### 2.6. Experimental Design and Statistical Analysis

A three-level, two-factor (3^2^) full factorial design (FFD) was conducted to investigate the extent of the decrease in DPV signal ascribable to ascorbic acid following heat treatment.

Two independent variables as heat treatment parameters for orange juice and three coded levels (−1, 0, and +1) were used as follows: X1 (temperature, °C) 70, 80, and 90°; X2 (thermal treatment time, min) 15, 45, and 60 min, whereas the dependent variable (response variable) was the voltammetric signal of AA. These values are significantly higher than those involved in conventional thermal processing methods in the food industry. However, as mentioned in the introduction, this work represents the initial phase of a study aimed at validating the proposed sensor for a rapid and cost-effective measurement of vitamin C content during food processing. Following recommendations of the literature [30,31,32], these combinations of time and temperature were chosen to ensure the detection of significant variations in vitamin C levels.

The signal decrease was calculated and expressed as a percentage by comparing the ascorbic acid measurements after heat treatment in the different conditions with the signal recorded by analyzing the untreated sample.

Control experiments were conducted concurrently for the same durations without applying heating. The percentage decrease values attributable to heat treatment were then adjusted to account for the values obtained from the control experiments. Each experiment was conducted in triplicate and executed in a randomized order to minimize systematic bias.

A two-way ANOVA with Bonferroni post hoc test was carried out to assess the significance of the two factors (time and temperature) and their interactions. The resulting dataset was then used to develop a predictive model through multiple linear regression (MLR) using the backward elimination criterion to select significant coefficients (*p* < 0.05).

All statistical calculations were performed using the Statistics for Data Analysis V.28 software package (SPS Srl, Bologna, Italy).

## 3. Results and Discussion

### 3.1. Characteristics of the Electrochemical GNP-PEDOT-Based Sensor

The characteristics of GNP-PEDOT-modified SPCEs were leveraged for rapid and reliable determination of vitamin C in the investigated food matrix based on the excellent electrocatalytic activity exhibited by PEDOT in the oxidation of ascorbic acid [33,34].

**Figure 1:**
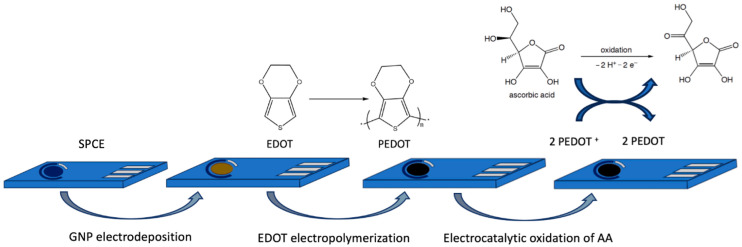
Scheme of the functionalization of SPCEs with GNP-PEDOT nanocomposite.

In particular, the developed sensors are based on cost-effective SPCEs modified with a nanocomposite material obtained by combining a layer of electrodeposited gold nanoparticles and electropolymerized PEDOT. As illustrated in Figure 1, the nanogold layer acts as a surface enhancer to activate the electrocatalytic process in which PEDOT plays the role of a redox mediator for the electrochemical oxidation of AA.

To fully exploit the unique properties of GNPs, their activation after deposition can be carried out rapidly and effectively by subsequent dipping in 1.2 M NaOH and 1.2 M HCl for a total treatment time of 10 min, following a procedure already tested in our recent study [28]. More specifically, the activation of gold nanoparticles based on the combined use of NaOH and HCl is a multifaceted process that affects their electrochemical properties, morphology, and catalytic activity. In fact, the voltammetric profiles of gold nanoparticles are significantly changed upon transition to an alkaline medium, showing a shift in redox activity and the formation of gold oxides [35]. On the other hand, the acidic environment provided by HCl induces the dissolution of gold oxides and the stabilization of the resulting GNPs, leading to improved surface chemistry [36].

**Figure 2:**
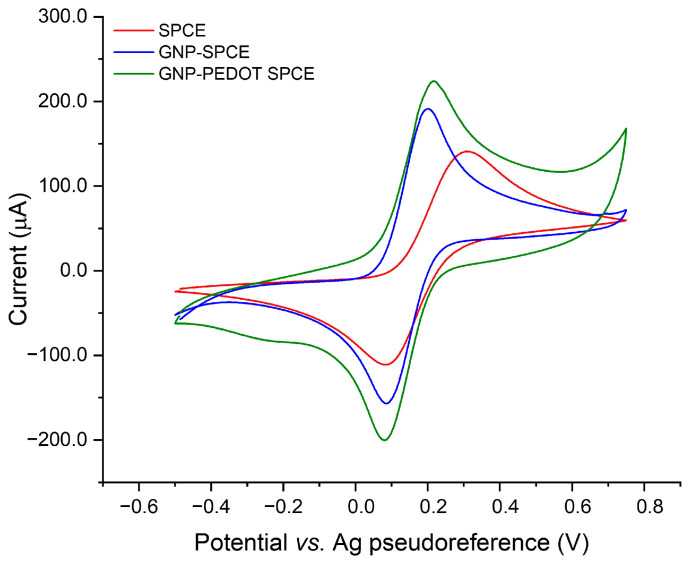
Stepwise CV characterization of bare SPCE, GNP-functionalized SPCE, and GNP-PEDOT-modified SPCE, carried out in 1 mM potassium ferrocyanide in PBS.

The SPCE electrode substrates were characterized after each modification step by cyclic voltammetry carried out using 1 mM ferrocyanide as a redox probe in PBS solution. The stepwise CV characterization is shown in Figure 2. A comparison of the voltammograms acquired after each step of the functionalization of SPCEs surface demonstrates a progressive increase in the electron transfer-related currents of the redox probe. This behavior can be ascribed to the increased surface area and electrocatalytic properties of GNPs.

In the subsequent step of PEDOT electropolymerization, a further change in the voltammetric pattern is observed, with both the forward and backward peaks undergoing an enhancement that can be ascribed to an additive effect of the electrochemical doping and undoping currents of the conducting polymer. A moderate shift of the peak potentials is also observed, which can be explained in terms of the redox mediation properties of PEDOT. Overall, these data provide evidence of the effective electrodeposition of the hybrid nanocomposite, as well as the redox activity of the conducting polymer.

**Figure 3:**
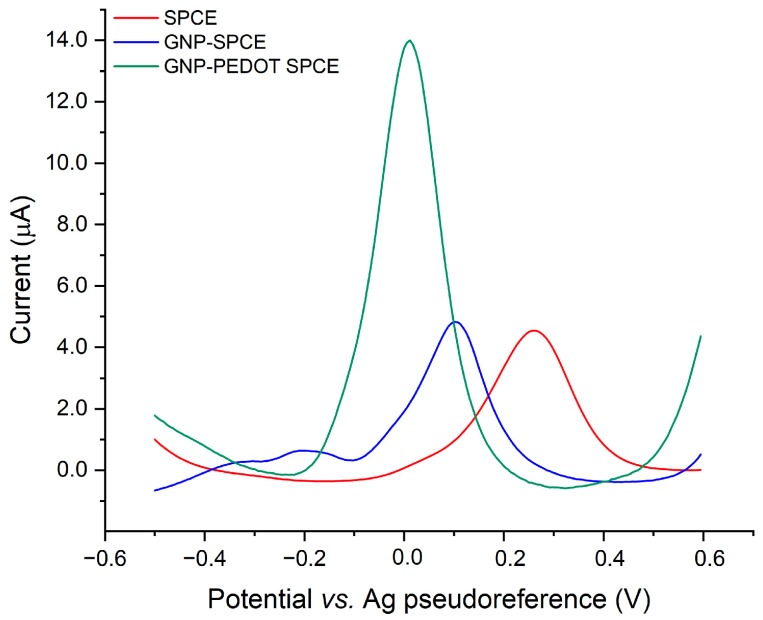
Comparison of the DPV responses for a 250 mg/L standard solution of ascorbic acid on bare SPCE, GNP-functionalized SPCE, and GNP-PEDOT-modified SPCE.

The remarkable electrocatalytic activity of PEDOT is demonstrated by a negatively shifted oxidation peak of AA (250 mg/L) and a significant enhancement in the anodic peak current compared to bare SPCEs and GNP-SPCEs, as reported in Figure 3.

It is worth noting that real-time voltammetric methods based on the use of unmodified SPCEs and SPCEs modified with platinum membranes and multi-walled carbon nanotubes have been recently proposed for the rapid and cost-effective testing of vitamin C in multifruit juices [22]. However, limitations in terms of matrix interferences attributable to anthocyanins were recognized by López-Pastor et al. [22]. This behavior can be explained by observing that the aforementioned screen-printed electrodes require the application of high voltages, up to +0.6 V, for the detection of vitamin C by cyclic voltammetry.

Recently, a strategy for the determination of ascorbic acid in food matrices based on the use of SPCEs modified with GNP and reduced graphene oxide (rGO) has been proposed [37]. Although the method has been shown to have good analytical performance, the fabrication process of such hybrid nanocomposites based on rGO is more laborious and time-consuming than PEDOT. Furthermore, considering that the electrochemical detection of ascorbic acid takes place at +0.24 V, interference from anthocyanins may also occur in this case for the detection in fruit juices. A similar approach based on the implementation of rGO and GNP as hybrid nanocomposites on indium tin oxide electrodes has been proposed by Mazzara et al. [38]. Simultaneous detection of uric acid and ascorbic acid was performed in different matrices, including fruit juice. However, the use of the more expensive indium tin oxide electrodes did not result in improved analytical performance compared to the more cost-effective approach proposed in our study.

In contrast, the electrocatalytic properties of PEDOT enable the detection of AA by DPV at +0.06 V, thus overcoming the limitations associated with its determination on non-functionalized electrodes with redox-active conducting polymers. Additionally, these constraints also include electrode fouling resulting from oxidation products and the need for a high oxidation potential of AA, which can lead to interferences and poor selectivity in complex matrices such as food samples [28]. The distinctive features of the GNP-PEDOT nanocomposite sensor are crucial for its effective application in the on-line monitoring of AA content during orange juice thermal treatment, particularly when compared to the use of biosensors based on enzymatic receptors. Despite the high performance for the determination of AA, biosensors undeniably present limitations, particularly regarding the stability of immobilized bioreceptors. Their usage necessitates controlled storage conditions in terms of temperature and humidity [39], and they are also generally expensive. It is important to note that the use of biosensors is essential in matrices such as biological fluids, where enzymatic mediation, such as by ascorbate oxidase, is required to eliminate interferences from compounds like dopamine and uric acid [27]. In the context of food matrices monitoring, the implementation of nanocomposites based on nanoparticles and redox-active conducting polymers, such as GNP/PEDOT electrodeposited on screen-printed carbon platforms, provides an optimal balance between performance and cost. The proposed sensing approach combines rapid and simple preparation, cost-effective materials, and the high stability of the sensing coating, allowing for storage in non-climate-controlled environments and significantly extended shelf-life.

### 3.2. Analytical Performance of GNP/PEDOT-Based Sensor for the Determination of Vitamin C

The analytical performance of the GNP/PEDOT-modified SPCE sensor was evaluated in standard ascorbic acid solutions in PBS, exploring a concentration range (45–600 mg/L) that matched the expected content of AA in fresh orange juice samples. Linearity across the entire explored range was demonstrated: the mean of the calculated regression residuals was found to be not significantly different from zero (*p* > 0.05).

**Figure 4:**
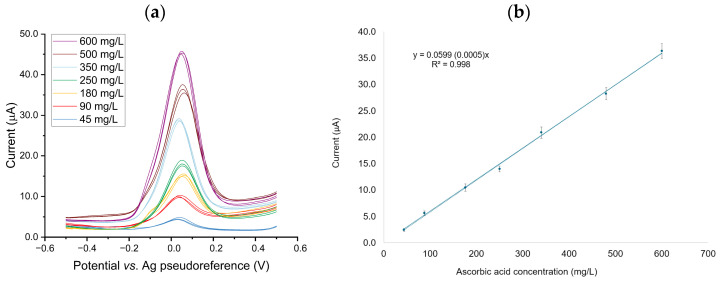
DPV scans recorded with the calibration standards over the 45–600 mg/L range (**a**) and corresponding calibration curve (**b**) with regression function in the inset; replicate measurements for each concentration level = 3.

DPV scans recorded with the calibration standards are reported in Figure 4a, whereas the corresponding calibration curve is shown in Figure 4b (calibration function inset).

The calculated LOD and LOQ values were 0.7 and 2.1 mg/L, respectively. Although the LOD values are higher than those reported in previous works [21,22], it is important to note that high sensitivity is not critical for this application since the concentrations of AA in fruit juices are several orders of magnitude higher than the calculated LOD values.

The intra-sensor repeatability was excellent across all levels of the calibration curve, giving an RSD below 5% (*n* = 9) assessed at low, medium, and high concentration ranges. Likewise, inter-sensor repeatability was excellent and fit the purpose: the replicate measurements carried out on three independent sensors gave RSD values below 10% (*n* = 3) at an AA concentration of 250 mg/L.

To assess the suitability of the DPV signal acquired using the GNP/PEDOT-based sensor to monitor the decrease in AA concentration due to heat treatment, a quantitative analysis was conducted on the untreated orange juice sample using the standard addition method. This approach estimated an AA content of 415 ± 9 mg/L. It is noteworthy that the excellent intra-sensor repeatability allowed all DPV scans to be acquired on a single GNP/PEDOT-modified SPCE sensor.

The selectivity of the sensor was evaluated by the addition of citric acid to the orange juice matrix. It was found that the sensor demonstrated excellent selectivity, even at concentrations of citric acid greater than 20 times the level of AA. The DPV signals were not significantly different (*p* > 0.05) from those of the blank sample, demonstrating that citric acid does not interfere with AA measurements (Figure S1).

Regarding the assessment of trueness, evaluated in terms of recovery rate, the measurement of the ascorbic acid concentration in a commercial orange juice sample with a claimed vitamin C content of 300 mg/L resulted in an estimated concentration of 283 ± 3 mg/L, corresponding to an excellent recovery rate *%RR* = 94 ± 1.

For shelf-life studies of the developed electrochemical sensor, it was demonstrated that the long-term stability of the electrodeposited GNP/PEDOT sensing layer ensures optimum sensor performance for at least two months. Weekly analyses of standard solutions containing 250 mg/L of ascorbic acid showed peak current values that were not significantly different (*p* < 0.05), validating the suitability of the sensor under non-temperature-controlled storage (Figure S2).

### 3.3. Experimental Design Applied to Heat Treatment and Predictive Modeling for Signal Reduction Assessment

**Figure 5:**
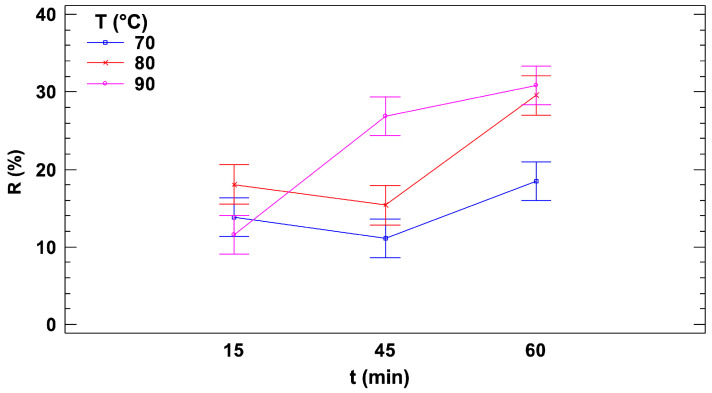
ANOVA interaction plot and 95 percent LSD intervals against treatment time for the three selected temperatures.

For each heat treatment condition, the percentage reduction of ascorbic acid was measured as a function of both time and temperature. To evaluate the analytical performance of the nanocomposite-based electrochemical sensor, a three-level, two-factor (3^2^) FFD was carried out, as described in Section 2.6. A two-way ANOVA with Bonferroni post hoc test confirmed the significance of the effects of time and temperature, as well as their interaction (*p* < 0.01), as illustrated in the interaction plot (Figure 5).

Figure 5 presents the signal reduction, R, along with confidence intervals calculated using Fisher’s Least Significant Difference (LSD) method, plotted against treatment time for the three selected temperatures. At the lowest temperature (70 °C), no significant differences among the three treatment times (15, 45, and 60 min) were observed due to overlapping confidence intervals. However, the overlap between the 60-min condition and the other two treatment times was minimal, suggesting a slight yet possibly meaningful increase in the reduction rate at 60 min. At 80 °C, no significant differences were detected between the two shorter treatment durations (15 and 45 min), but a substantial increase in the reduction rate was observed after 60 min, with a reduction increase of more than 50% compared to shorter treatment times. At 90 °C, a distinct behavior was observed compared to the other two temperatures. A significant increase in the reduction rate occurred between 15 and 45 min, marked by a steep slope, followed by a plateau between 45 and 60 min. This plateau may indicate the complete depletion of AA at the 45-min case, beyond which additional heating did not result in further reduction, even with prolonged treatment time.

Interestingly, for treatments lasting 15 min, no significant differences in reduction rates were observed across the three temperatures (70 °C, 80 °C, and 90 °C). In contrast, for 45-min treatments, 90 °C resulted in a significantly higher reduction rate compared to the lower temperatures. Similarly, at 60 min, 90 °C continued to produce the highest reduction rate, while 80 °C also showed a significant increase in reduction compared to 70 °C.

Concluding, the sensor has demonstrated its capability to detect variations in AA levels within the sample resulting from different heat treatments. This sensitivity allows it to effectively distinguish between the diverse conditions applied to the food matrix, underscoring its potential as a reliable analytical tool for monitoring the thermal degradation of AA in food processing.

**Figure 6:**
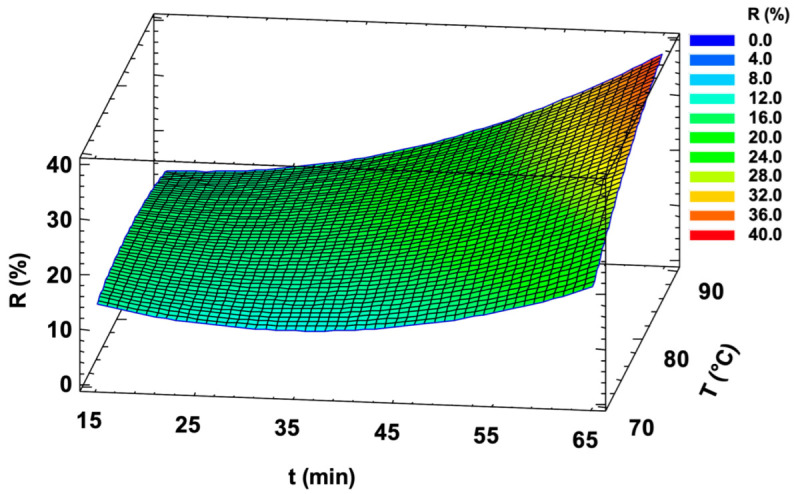
Response surface of the calculated predictive model for the decrease in ascorbic acid extent associated with heat treatment of orange juice samples.

Based on these findings, the dataset was processed by MLR to develop a predictive model for the effect of heat treatment on ascorbic acid retention in orange juice samples. The response function calculated by MLR is presented in Equation (1), while the corresponding response surface is illustrated in Figure 6.

(1) $$R\;\left(\%\right)=-97.269\left(9.149\right)-1.955\left(0.274\right)\cdot t+3.260\left(0.709\right)\cdot T+0.010\left(0.003\right)\cdot t^{2}+0.018(0.004)\cdot t\cdot T$$

being the signal reduction *R* (%) correlated to both time *t* (min) and to temperature *T* (°C).

The model provides a useful basis for defining treatment processes in order to achieve the ideal combination of food safety and final product quality. As might be easily assumed, it can be observed that combinations of low temperatures and short times ensure the best preservation of vitamin C. Furthermore, it suggests that below a certain threshold value, which appears to be around the combination of 40 min and 80 °C, the percentage reduction does not show significant increases, remaining at limited values.

## 4. Conclusions

The proposed electrochemical sensor for on-line monitoring of vitamin C in fruit juice in processing plants proved effective in detecting variations in ascorbic acid levels in samples subjected to different heat treatments, successfully distinguishing between the applied processing conditions. There was excellent precision in terms of both intra- and inter-sensor repeatability as well as long-term stability. These results highlight the potential of the sensor as a tool for on-line monitoring during pasteurization and sterilization of fruit juices, facilitating real-time adjustments of process parameters and improving final product quality.

In addition, the GNP/PEDOT-modified electrochemical sensor is potentially suitable for continuous in-line operation using chronoamperometric measurement at a constant potential, also exploiting the integration with smart and portable instrumentation [40]. Periodic regeneration cycles would be sufficient to restore the PEDOT to its reduced form.

Further evaluation under real-world operating conditions is necessary to integrate the proposed sensor into the production environment as an intelligent system. This would include electronics capable of storing vitamin C profiles in a controller and utilizing the collected data to optimize the production process over time.

## Data Availability

The raw data supporting the conclusions of this article will be made available by the authors upon request.

## Supplementary Materials

The following supporting information can be downloaded at: https://www.mdpi.com/article/10.3390/s25051385/s1, Figure S1: Selectivity study of GNP-PEDOT-modified SPCEs: DPV scans acquired by analyzing the untreated orange juice sample and after two successive additions of citric acid standard solution with a concentration 20 times higher than that used for the quantification of ascorbic acid; Figure S2: Shelf-life study of GNP-PEDOT-modified SPCE: average values and standard deviations (n = 3) of DPV peak currents measured by analyzing 250 mg/L standard solutions at seven days intervals for 2 months.

## Abbreviations

The following abbreviations are used in this manuscript:

3H2P	3-Hydroxy-2-Pyrone
AA	Ascorbic Acid
DPV	Differential Pulse Voltammetry
EDOT	3,4-EthyleneDiOxyThiophene
FFD	Full Factorial Design
GNP	Gold NanoParticles
LOD	Limit Of Detection
LOQ	Limit Of Quantification
PAT	Process Analytical Technology
PBS	Phosphate-Buffered Saline
PEDOT	Poly(3,4-EthyleneDiOxyThiophene)
RR	Recovery Rate
SPCE	Screen-Printed Carbon Electrode
