# Supplementary material for: On-Line Monitoring of Vitamin C in Fruit Juice in Processing Plants by Electrochemical Sensor Based on PEDOT-Modified Electrodes: A Feasibility Study
Source: Sensors (Basel). 2025 Feb 24;25(5):1385. doi: 10.3390/s25051385 (PMC11902688; doi:10.3390/s25051385)
Supplement: Supplementary file 1 [file sensors-25-01385-s001.zip › sensors-3464539-supplementary.pdf]

Supplementary Material

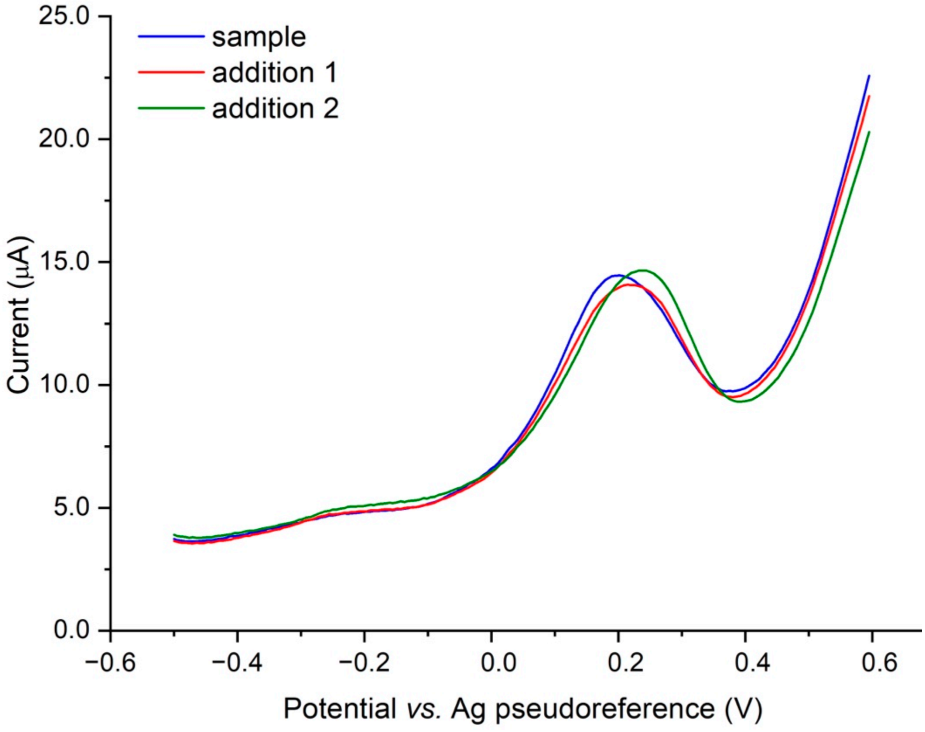

Figure S1. Selectivity study of GNP-PEDOT-modified SPCEs: DPV scans acquired by analysing the untreated orange juice sample and after two successive additions of citric acid standard solution with a concentration 20 times higher than that used for the quantification of ascorbic acid.

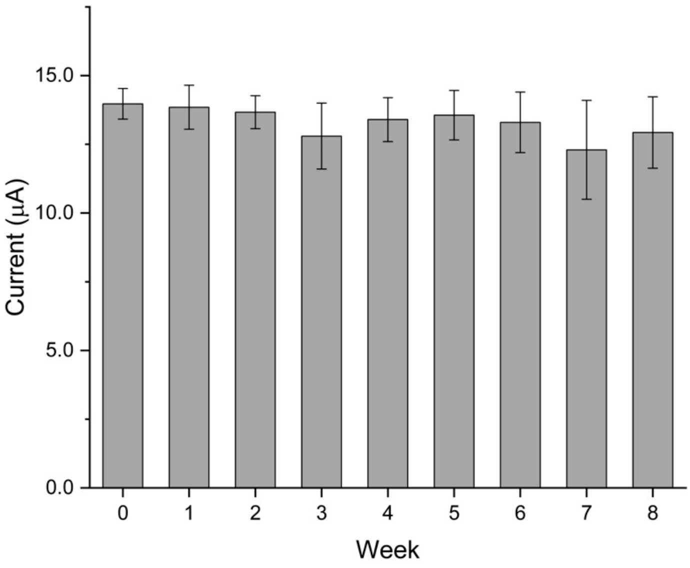

Figure S2. Shelf-life study of GNP-PEDOT-modified SPCE: average values and standard deviations (*n*=*3*) of DPV peak currents measured by analyzing 250 mg/L standard solutions at seven days intervals for 2 months.
